# Rap1‐mediated nucleosome displacement can regulate gene expression in senescent cells without impacting the pace of senescence

**DOI:** 10.1111/acel.13061

**Published:** 2019-11-19

**Authors:** Shufei Song, Javier V. Perez, William Svitko, M. Daniel Ricketts, Elliot Dean, David Schultz, Ronen Marmorstein, F. Brad Johnson

**Affiliations:** ^1^ Department of Biochemistry and Molecular Biophysics University of Pennsylvania Philadelphia PA USA; ^2^ Graduate Group in Biochemistry and Molecular Biophysics University of Pennsylvania Philadelphia PA USA; ^3^ Department of Pathology and Laboratory Medicine University of Pennsylvania Philadelphia PA USA; ^4^ Abramson Family Cancer Research Institute University of Pennsylvania Philadelphia PA USA; ^5^ High‐Throughput Screening Core University of Pennsylvania Philadelphia PA USA; ^6^ Institute on Aging University of Pennsylvania Philadelphia PA USA

**Keywords:** cellular senescence, pace of senescence, pioneer transcription factor, Rap1

## Abstract

Cell senescence is accompanied, and in part mediated, by changes in chromatin, including histone losses, but underlying mechanisms are not well understood. We reported previously that during yeast cell senescence driven by telomere shortening, the telomeric protein Rap1 plays a major role in reprogramming gene expression by relocalizing hundreds of new target genes (called NRTS, for **n**ew **R**ap1 **t**argets at **s**enescence) to the promoters. This leads to two types of histone loss: Rap1 lowers histone level globally by repressing histone gene expression, and it also causes local nucleosome displacement at the promoters of upregulated NRTS. Here, we present evidence of direct binding between Rap1 and histone H3/H4 heterotetramers, and map amino acids involved in the interaction within the Rap1 SANT domain to amino acids 392–394 (SHY). Introduction of a point mutation within the native *RAP1* locus that converts these residues to alanines (*RAP1^SHY^*), and thus disrupts Rap1‐H3/H4 interaction, does not interfere with Rap1 relocalization to NRTS at senescence, but prevents full nucleosome displacement and gene upregulation, indicating direct Rap1‐H3/H4 contacts are involved in nucleosome displacement. Consistent with this, the histone H3/H4 chaperone Asf1 is similarly unnecessary for Rap1 localization to NRTS but is required for full Rap1‐mediated nucleosome displacement and gene activation. Remarkably, *RAP1^SHY^* does not affect the pace of senescence‐related cell cycle arrest, indicating that some changes in gene expression at senescence are not coupled to this arrest.

## Graphical abstract

Rap1 interacts directly with histones to displace nucleosomes at the promoters of upregulated genes at senescence. A mutation that perturbs Rap1‐histone interactions leads to compromised nucleosome loss and blunted gene activation, but no change is observed in the rate of senescence. Our findings shed light on mechanisms of Rap1‐mediated gene expression changes at senescence and show that these can be uncoupled from the pace of senescence.

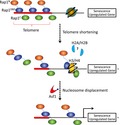

## 1 INTRODUCTION

Cellular senescence is a programmed response to stresses that put cells at risk for becoming cancerous, through events such as DNA damage, telomere and mitochondrial dysfunction, and oxidative stress. It is characterized by stable cell cycle arrest, but importantly also by profound alterations in chromatin structure leading to changes in gene expression that impact cell metabolism and the secretion of factors that influence the function of tissues in which senescent cells reside (Campisi, 2013; Ritschka et al., 2017; Sapieha & Mallette, 2018; van Deursen, 2014). Although cell senescence plays beneficial roles early in life by contributing to tumor suppression, wound healing, and immunity, several lines of evidence suggest that it can also drive age‐related pathologies through stem cell depletion (Krishnamurthy et al., 2006; Molofsky et al., 2006) or by disruption of tissue structure and function, apparently via secretion of factors such as proteases and inflammatory cytokines (Baar et al., 2017; Baker et al., 2016, 2011; Childs et al., 2016; Schafer et al., 2017). Understanding the mechanisms underlying cell senescence, particularly those regulating altered gene expression, is thus of substantial interest.

One important driver of human cellular senescence is telomere shortening. Critically short (i.e., “uncapped”) telomeres are recognized by the DNA damage response (DDR) machinery, leading to arrest and gene expression changes. Senescence driven by telomere shortening can be modeled in *Saccharomyces cerevisiae*. Yeast naturally expresses telomerase to maintain telomere length, but if telomerase is inactivated genetically, cells gradually lose telomeric DNA through rounds of division and eventually arrest—although rare survivors, which maintain telomeres via homologous recombination, eventually emerge from senescent populations. Many factors known to influence senescence in human cells have similar roles in telomerase‐deficient yeast, including exonucleases, helicases, and DDR proteins (Herbig, Jobling, Chen, Chen, & Sedivy, 2004; IJpma & Greider, 2003; Johnson et al., 2001; Ritchie, Mallory, & Petes, 1999; Schaetzlein et al., 2007).

A key and conserved feature of senescence, and other types of aging‐related biology, from yeast to humans is histone loss (Ivanov et al., 2013; Liu et al., 2013; O'Sullivan & Karlseder, 2012; Platt et al., 2013; Song & Johnson, 2018). Histone gene expression, global levels of all core histones, and nucleosome occupancy at particular genomic sites are all decreased in senescent telomerase‐deficient yeast (Platt et al., 2013), and similar observations have been made in aged yeast mother cells (Feser et al., 2010; Hu et al., 2014). This loss is apparently closely linked to the altered gene expression observed in senescent cells, as highly similar gene expression patterns are seen when histone levels are artificially downregulated (Platt et al., 2013). In both yeast models, artificial overexpression of core histones promotes longevity. However, little is known about the mechanisms underlying histone‐related changes in senescent cells.

Previously, we found that the telomeric protein Rap1 plays a major role in replicative senescence in telomerase‐deficient yeast, including regulation of histone gene expression and site‐specific nucleosome occupancy (Platt et al., 2013). Rap1 is conserved between yeast and humans, and the yeast protein binds directly to telomere repeat DNA in a sequence‐specific fashion via two tandemly arranged Myb domains, where it plays roles in regulating telomere length, transcriptional silencing, and capping (Kyrion, Liu, Liu, & Lustig, 1993; Marcand, Wotton, Gilson, & Shore, 1997; Martínez, Gómez‐López, Pisano, Flores, & Blasco, 2016; Moretti & Shore, 2001; Pardo & Marcand, 2005; Rai, Chen, Lei, & Chang, 2016; Vodenicharov, Laterreur, & Wellinger, 2010; Yang et al., 2017). It also functions to regulate transcription throughout the genome, in particular repressing expression of the silent mating‐type loci, and activating expression of approximately ten percent of all yeast genes, particularly the highly expressed ribosomal protein and certain glycolytic enzyme genes. During replicative senescence, Rap1 relocalizes from shortened telomeres and subtelomeres to the promoters of hundreds of new genes, named NRTS (**n**ew **R**ap1 **t**argets at **s**enescence), which have lower affinity Rap1 binding sites than natural Rap1 targets. Among the NRTS are the genes that encode the core histone proteins, which are transcriptionally repressed by Rap1, thus contributing to the loss of histone proteins observed at senescence. In contrast to the histone genes, the majority of NRTS become activated by Rap1. This activation is associated with the displacement by Rap1 of nucleosomes from the promoters of these NRTS, but it is not known if nucleosome displacement causes NRTS activation (Platt et al., 2013). Furthermore, Rap1 drives the overall pace of senescence, because it is delayed by experimental diminishment of Rap1 levels. However, whether the function of Rap1 to repress global histone levels, or its function to locally displace nucleosomes and upregulate NRTS, might underlie its effect on the rate of senescence has not been tested.

It has long been known that Rap1 can bind nucleosomal DNA, and its ability to exclude nucleosomes from promoters is functionally similar to pioneer transcription factors (pTFs) in higher eukaryotes (Ganapathi et al., 2011; Knight et al., 2014; Koerber, Rhee, Jiang, & Pugh, 2009; Kubik et al., 2015; Lickwar, Mueller, Hanlon, McNally, & Lieb, 2012; Rhee & Pugh, 2011; Yan, Chen, & Bai, 2018; Yarragudi, Miyake, Li, & Morse, 2004; Yu, Sabet, Chambers, & Morse, 2001; Zaret & Carroll, 2011). However, the mechanisms by which Rap1 displaces histones have not been thoroughly explored. It is possible that direct contacts between Rap1 and histones are involved, because proteome‐wide interaction screens in yeast and genome‐wide split‐YFP complementation assays in human cells suggest that Rap1 proteins may bind histones (Gilmore et al., 2012; Lee et al., 2011), although this has not been studied in detail. This possibility is of general interest because histone binding has been so far described for only two other pTFs, FoxO1 and FoxA (Cirillo et al., 2002; Hatta & Cirillo, 2007). In addition, we reasoned that if Rap1‐histone contacts are involved in histone displacement by locally bound Rap1, but not in other Rap1 functions including histone gene repression, then a Rap1 mutant selectively deficient in histone contact would provide a tool to not only address the role of nucleosome displacement in NRTS activation, but also test the importance of NRTS upregulation in driving the rate of senescence.

Here we describe the creation of such a Rap1 mutant, identified based on its disruption of a direct interaction established between Rap1 and histone H3/H4 heterotetramers, as well as the effects of the mutation on the functions of Rap1 at senescence. We also describe a role for a histone H3/H4 chaperone, Asf1, in nucleosome displacement and NRTS upregulation by Rap1.

## 2 RESULTS

### 2.1 Rap1 binds H3/H4 histone tetramers

As reviewed above, Rap1 is a nucleosome‐displacing factor, functionally similar to pTFs in higher eukaryotes. Consistent with this function of Rap1, we previously reported that Rap1 displaces nucleosomes from the promoters of activated NRTS at senescence. In contemplating underlying mechanisms, we considered evidence indicating potentially direct binding between histones and the yeast and human Rap1 proteins. This evidence comes from a proteome‐wide screen in yeast and a genome‐wide split‐YFP fluorescence complementation screen in human cells (Gilmore et al., 2012; Lee et al., 2011), but the apparent Rap1‐histone interactions have not been investigated in any detail. Such an interaction, if mapped, could provide us with tools to manipulate Rap1 functions at senescence. Therefore, we decided to explore the possibility that nucleosome displacement by Rap1 might involve direct interactions with histone proteins.

**Figure 1:**
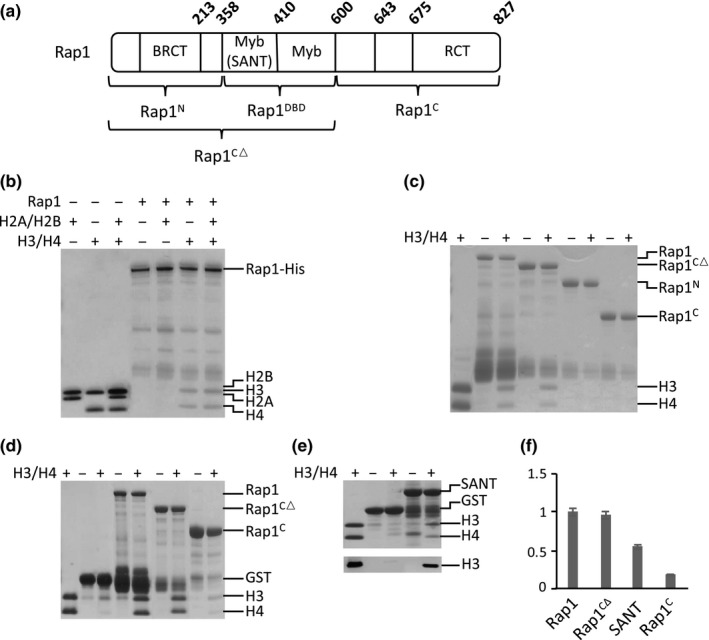
Rap1 binds histone H3/H4 tetramers. (a) Schematic of Rap1 fragments and domains, with amino acid positions indicated. (b) GST‐Rap1‐6X‐His protein was expressed in *E. coli*, purified using the His tag and then subjected to a GST pull‐down histone binding assay (300 mM NaCl; note that similar results were obtained up to at least 750 mM NaCl). Rap1 (0.5 μM) binds to H3/H4 tetramers (2 μM), but not H2A/H2B (2 μM) dimers. When H2A/H2B dimers and H3/H4 tetramers are mixed in 2:1 ratio as in the octameric histone core (4 μM H3/H4 and 2 μM H2A/H2B), Rap1 interacts specifically with H3/H4 tetramers. (c) Panels c–e use Rap1 protein purified using only the GST‐tag. GST pull‐down histone binding assay with truncated regions of Rap1 (400 mM NaCl) is shown. Rap1^CΔ^ (0.5 μM) binds H3/H4 tetramers (2 μM) with similar strength as full‐length Rap1. Rap1^N^ and Rap1^C^ do not bind histones under these conditions. Note that when Rap1 constructs are not purified via a C‐terminal 6X‐His tag, proteins near the size of GST are detected, presumably due to translational termination or proteolytic degradation near the GST‐Rap1 junction. (d) GST pull‐down histone assay with equimolar Rap1 and histones (300 mM NaCl). Full‐length Rap1 (2 μM) and Rap1^CΔ^ (2 μM) each binds H3/H4 (2 μM) robustly (~1:1). Rap1^C^ (2 μM) displays detectable H3/H4 binding under these conditions. Similar interaction strengths are observed from salt concentrations ranging from 150 to 750 mM NaCl. (e) GST pull‐down histone binding assay with SANT domain (300 mM NaCl). Top panel: Coomassie stain of SANT domain (2 μM) interacting with H3/H4 (2 μM). Bottom panel: immunoblot against histone H3. (f) Quantitation of Rap1 fragments binding to H3/H4 under equimolar conditions. H3 signal is normalized to loading control, with full‐length wildtype Rap1 binding strength set as 1.0.  Error bars indicate the standard error of the mean (*N* = 4)

To verify Rap1‐histone binding and begin to map the Rap1 regions involved, we fused a GST‐tag to the N‐terminus of full‐length Rap1 and to various fragments of the protein (Figure 1a). These comprise an N‐terminal fragment (amino acids 1–358, Rap1^N^), which contains a BRCT domain, and can be deleted with little to no effect on cell growth and gene transcription (Mizuno et al., 2004; Shore, 1994); the DNA binding domain (amino acids 359–600, Rap1^DBD^), which consists of two tandem Myb domains, the first of which is also a SANT domain; and a C‐terminal fragment (amino acids 601–827, Rap1^C^) containing the RCT domain, which interacts with various Rap1 binding partners including Rif1, Rif2, Sir3, and Sir4 (Moretti & Shore, 2001; Shi et al., 2013; Strahl‐Bolsinger, Hecht, Luo, & Grunstein, 1997).

We performed the histone binding assay by incubating 0.5 μM of GST‐tagged Rap1 proteins bound to glutathione beads with 2 μM H2A/H2B dimers or H3/H4 tetramers. The beads were then washed under stringent conditions, and retained histones were examined by SDS‐PAGE. We found that Rap1 bound to H3/H4 tetramers, but not H2A/H2B dimers, including under conditions where the H3/H4 and H2A/H2B proteins were mixed with one another prior to binding (Figure 1b). Significant binding was observed in salt concentrations ranging from 150 to 750 mM. Rap1^N^ did not bind to H3/H4, whereas the Rap1^DBD^ showed similar binding strength compared to full‐length Rap1 (Figure 1c). When increased to levels equimolar to the histones (2 μM each), full‐length Rap1 and Rap1^CΔ^ showed more robust binding to H3/H4 tetramers, whereas the C‐terminal fragment also displayed weak binding (Figure 1d,f). However, even in 7.5‐fold molar excess, Rap1^N^ failed to bind histones (Figure S1). Taken together, our findings indicate Rap1 interacts directly with the H3/H4 histone tetramer, which involves relatively strong versus weak binding interactions between histones and the Rap1 DBD versus C‐terminus.

The Rap1 DBD consists of two tandem Myb domains, the first of which is also classified as a SANT domain (amino acids 360–410). The SANT domain is a stretch of approximately 50 amino acids containing the helix‐turn‐helix motif and is typically involved in protein–protein interactions. Some SANT domains can bind to histone tails and have been proposed to function as histone interaction modules important for nucleosome remodeling (Boyer et al., 2002; Boyer, Latek, & Peterson, 2004; Grüne et al., 2003). Therefore, we predicted that the H3/H4 interaction seen in the DBD involves interaction surfaces within the SANT domain. To test whether it might be sufficient for binding, we fused GST to the Rap1 SANT domain and confirmed that it binds the H3/H4 tetramers under equimolar concentrations (2 μM each), with approximately half the affinity of the full‐length protein (Figure 1e,f). These findings indicate that the SANT domain contributes substantially to the capacity of Rap1 to bind histone H3/H4 tetramers.

### 2.2 Amino acids 392–394 (SHY) facilitates Rap1‐histone interactions

**Figure 2:**
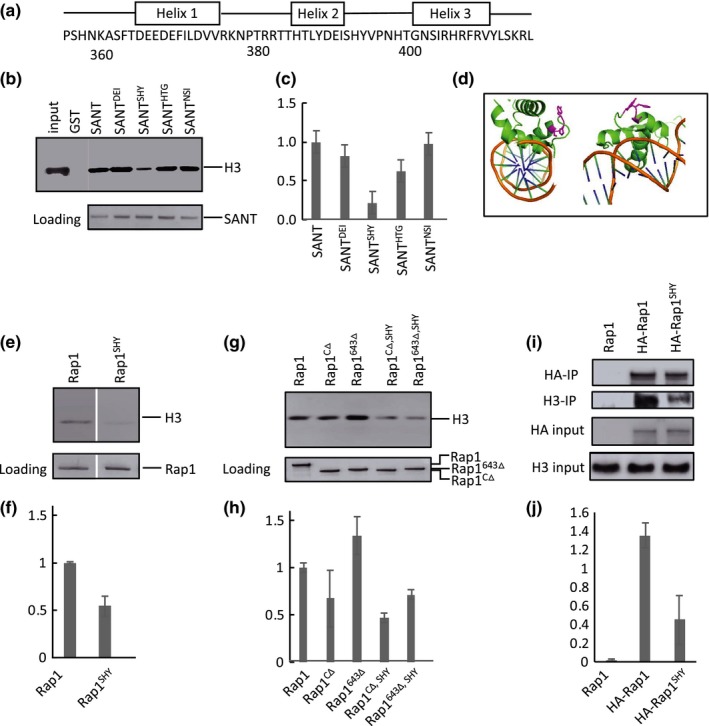
Amino acids 392–394 (SHY) facilitate Rap1‐histone interactions. (a) Location of alpha‐helices within the SANT domain, redrawn from Konig et al. (1996). Triple alanine mutants were generated from amino acids 359–410. (b) Immunoblot analysis of in vitro GST pull‐down assay of histones showing representative triple alanine mutants. Pull‐down was performed with equimolar GST‐SANT (0.5 μM) and histones (0.5 μM) at 400 mM NaCl. Bottom panel is the blot stained with Ponceau S as a loading control. (c) Quantitation of triple alanine mutants binding to H3/H4, normalized to Ponceau stain signal, and with WT SANT set to 1.0. Error bars for all quantitations indicate standard error of the mean (*N* = 2). Only mutant 12 (amino acids 392–394, SHY) showed a significant loss of H3 signal. (d) Two views of the SANT domain bound to DNA. Amino acids SHY side chains are colored in magenta. SHY is located immediately C‐terminal to helix 2, with side chains facing away from the Rap1‐DNA interaction surface. Image generated using Pymol (PDB ID: 3UKG). (e) Representative immunoblot analysis of GST pull‐down histone binding assay with full‐length Rap1 and Rap1^SHY^. Pull‐down was performed with 0.5 μM each Rap1 and H3/H4 at 400 mM NaCl. Rap1^SHY^ displays a ~50% loss of histone binding. Bottom panel is a loading control gel stained with Coomassie blue. (f) Quantitation of full‐length Rap1 and Rap1^SHY^ binding to H3/H4 (*N* = 3). (g) Representative immunoblot analysis of GST pull‐down histone assay using two truncated versions of Rap1 lacking the C‐terminus, Rap1^CΔ^ and Rap1^643 Δ^. Pull‐down was performed with 2 μM Rap1 truncated constructs and 2 μM H3/H4 at 400 mM NaCl. Both truncated forms show a significant and similar loss of histone signal when amino acid SHY is mutated to AAA (rightmost two lanes). Bottom panel is Coomassie loading control. (h) Quantitation of g (*N* = 3). (i) Representative coimmunoprecipitation of histone H3 with immunoprecipitated HA‐Rap1 and HA‐Rap1^SHY^. Input is 5% of the WCE, and Rap1^SHY^ shows a significant loss of histone binding in the extracts. (j) Quantitation of the ratio of co‐immunoprecipated H3 to input H3 signals in i (*N* = 3)

To identify residues within the Rap1 SANT domain required for binding H3/H4 tetramers, we generated GST‐tagged triple alanine mutants in which consecutive blocks of three amino acids in the SANT domain are mutated to alanines (Figure 2a). Each of the mutants was separately purified and incubated with equimolar concentration of H3/H4 tetramers (0.5 μM). Elutions from the glutathione beads were analyzed by Western blotting using H3 antibodies. A significant loss of H3 signal was observed when amino acids 392 (serine), 393 (histidine), and 394 (tyrosine) were mutated (Figures 2b,c and S2). Based on X‐ray crystal structures of the Rap1 DBD bound to DNA (Konig, Giraldo, Chapman, & Rhodes, 1996; Matot et al., 2012), amino acids 392–394 are located in the turn between helix two and three. This turn faces away from the Rap1‐DNA interaction surface (Figure 2d), consistent with potential involvement of the SHY patch in interactions between DNA‐bound Rap1 and other proteins.

We next tested to see if replacement of SHY by AAA in longer stretches of Rap1 yielded similar loss of histone binding. Full‐length Rap1 containing the replacement (Rap1^SHY^) showed a 50% loss of H3/H4 binding signal (Figure 2e,f). Similar losses were observed when the SHY to AAA replacement was introduced into Rap1 lacking the entire C‐terminal fragment (Rap1^CΔ^), or a portion of it (Rap1^643Δ^) (Figure 2g,h; see Figure 1a for map). This is consistent with an only minor role for the C‐terminus in stabilizing Rap1‐histone interactions.

To address whether the SHY patch impacts binding of Rap1 to soluble histones in vivo, we immunoprecipitated HA‐tagged Rap1 and Rap1^SHY^ from whole‐cell extracts (WCE) and immunoblotted for H3. The extracts were treated with benzonase to prevent indirect DNA‐mediated interactions between the proteins. Rap1^SHY^ showed a significant loss of H3 binding compared to WT Rap1 (Figure 2i,j). Together with the in vitro pull‐down data, this strongly supports a physical interaction between Rap1 and H3/H4 that involves amino acids in the SHY patch of the DNA binding domain.

### 2.3 Rap1^SHY^ is deficient in NRTS activation and histone displacement

**Figure 3:**
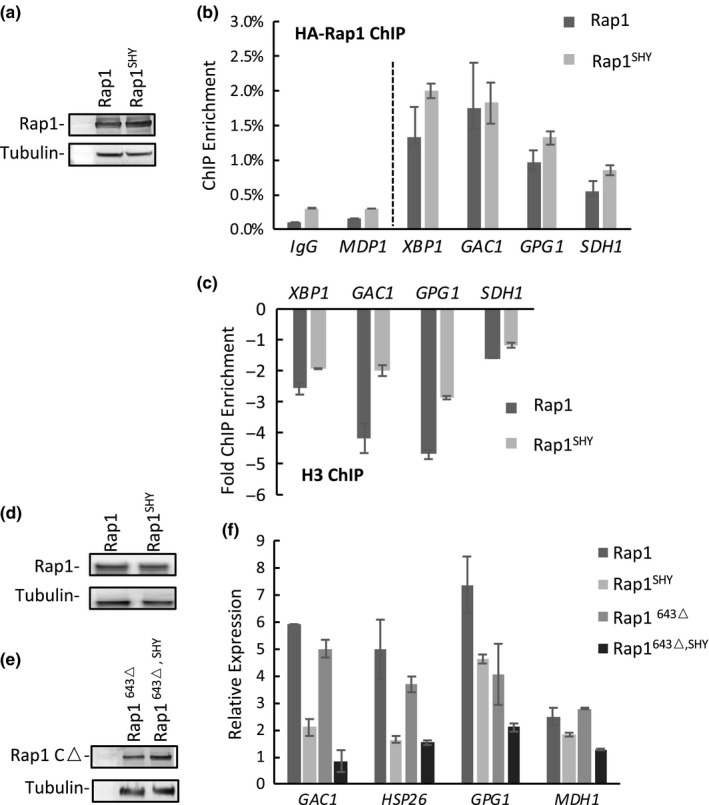
Rap1^SHY^ is deficient in NRTS activation and histone displacement. (a) Immunoblot analysis of TCA extracts of *GAL1‐*driven HA‐Rap1 and HA‐Rap1^SHY^ accumulation after 130 min of induction with galactose, conditions also used for panels (b) and (c). (b) Rap1 levels at the promoters of the upregulated NRTS, measured by qPCR of ChIP samples from cells overexpressing HA‐Rap1 or HA‐Rap1^SHY^, and normalized to input. IgG is control immunoglobulin from nonimmunized rabbit, and *MDP1* is a non‐Rap1 target. (c) Loss of H3 levels at the promoters of the upregulated NRTS. The fold H3 ChIP enrichment is the ratio of H3 levels at the promoters of the activated NRTS in induced versus uninduced cells, normalized to their levels at the promoter of the non‐Rap1 target gene *MDP1* (*p* < .04). (d) Accumulation of HA‐Rap1 and HA‐Rap1^SHY^ driven by the *NOP1* promoter. (e) Accumulation of HA‐Rap1^CΔ^ and HA‐Rap1^CΔ,SHY^ driven by the *NOP1* promoter. (f) mRNA levels of activated NRTS induced by Rap1 overexpression, measured by qPCR, and normalized to *ACT1* and vector control. Rap1^SHY^ and Rap1^643Δ,SHY^ are similarly compromised in NRTS activation (*p* < .03). All error bars indicate the standard error of the mean

Given the physical interactions observed between Rap1 and H3/H4 tetramers, we proceeded to investigate the functional effects of Rap1^SHY^ in vivo, in particular its effects on the different functions of Rap1 at senescence. We hypothesized that the compromised ability of Rap1^SHY^ to interact with the H3/H4 tetrameric core of nucleosomes would interfere with its roles in NRTS promoter clearance and gene activation. As the SHY to AAA mutation is within the DNA binding domain, we first confirmed that Rap1^SHY^ did not compromise the ability of Rap1 to bind DNA. Electrophoretic mobility shift assays (EMSA) using a telomeric sequence, a natural Rap1 binding site within the *TEF2* promoter, and a representative NRTS promoter demonstrated Rap1 and Rap1^SHY^ bound DNA with similar affinities (Figure S3a–c). We next used a system of Rap1 overexpression in wild‐type cells, which we showed previously recapitulates the selective binding of Rap1 to NRTS promoters, from which nucleosomes are displaced and gene expression is upregulated. Wild‐type cells were transformed with 2‐micron based plasmids from which either HA‐tagged Rap1 or Rap1^SHY^ expression is driven by the *GAL1* promoter. Expression was induced with galactose for 130 min, which we reported previously is sufficient for local histone displacement at promoters by Rap1 but avoids potential secondary effects from toxicity manifesting as growth inhibition after eight hours of induction (Platt et al., 2013). Rap1 localization to NRTS promoters and histone displacement were measured by ChIP‐qPCR, using antibodies against the HA‐tag and H3, respectively. Total cellular levels (Figure 3a) and localization to NRTS promoters (Figure 3b) were similar for both proteins, consistent with their similar DNA binding abilities. However, Rap1^SHY^ did not displace nucleosomes as efficiently compared to WT (Figure 3c; see also Figure S3d, demonstrating greater histone H3 losses from the ChIPed promoters following induction of Rap1 vs. Rap1^SHY^). To test whether compromised nucleosome displacement resulted in changes in gene expression, we constitutively expressed full‐length Rap1 and a C‐terminally truncated version of Rap1 (Rap1^643Δ^) and their respective SHY to AAA mutants, from a 2‐micron plasmid driven by the *NOP1* promoter, a nontoxic Rap1 overexpression system which has been previously shown to be sufficient for elevated NRTS expression. Consistent with the reduced levels of H3 displacement seen by ChIP, Rap^SHY^ does not activate NRTS mRNA expression as strongly as WT (Figure 3f). Rap1^643Δ^ can also upregulate NRTS expression, though to a slightly lower level compared to full‐length Rap1, consistent with a role for both the SANT and C‐terminus in histone interactions (Figure 3f). Much like Rap1 and Rap1^SHY^, a similar decrease in NRTS expression was observed in Rap1^643Δ, SHY^ compared to Rap1^643Δ^ (Figure 3f). However, no changes in expression were observed for representative natural Rap1 target genes, including the glycolytic gene *ENO2* and the ribosomal protein gene *RPS5*, nor for non‐Rap1 targets, when comparing strains overexpressing the WT and mutant proteins (Figure S3e,f). These findings suggest that Rap1‐histone interactions involving the SHY patch and C‐terminal region are not required for binding to NRTS promoters but contribute to Rap1‐mediated nucleosome displacement and NRTS activation.

### 2.4 Rap1^SHY^ confers diminished NRTS activation at senescence without affecting the rate of senescence

To examine the effects of Rap1^SHY^ in the context of senescence, we introduced the SHY to AAA mutation within one of the endogenous *RAP1* loci in a *TLC1/tlc1*Δ diploid. Upon sporulation and dissection of tetrads, the Rap1^SHY^ haploid spore products formed smaller colonies (Figure S4a). Southern blot analysis using probes for Y’ telomeric fragments showed similar telomeric lengths in *RAP1* and *RAP1^SHY^* strains, as well as in their respective telomerase deletion (*tlc1Δ*) strains at 50 population doublings after spore germination (Figure S4b), suggesting a normal level of telomere capping and maintenance by Rap1^SHY^. This is consistent with the similar colony sizes observed for *RAP1^SHY^ tlc1Δ* double mutants and *tlc1Δ* strains, at least for the ~20–25 divisions needed for colony formation from the germinated spores (Figure S4a). Furthermore, the colony‐forming efficiency of isolated *RAP1^SHY^* cells is similar to WT (Figure S4c), implying that slow growth is not due to increased cell death. Expression of the natural Rap1 target genes *ENO2* and *RPS5*, encoding glycolytic and ribosomal proteins, respectively, are not diminished by the SHY mutation (Figures S4g and S3d,e), and so we do not yet have an explanation for the slow growth of the mutants. In addition, Rap1^SHY^ functions normally to silence subtelomeres (Figure S4d,e) and the silent mating‐type loci (Figure S4f).

**Figure 4:**
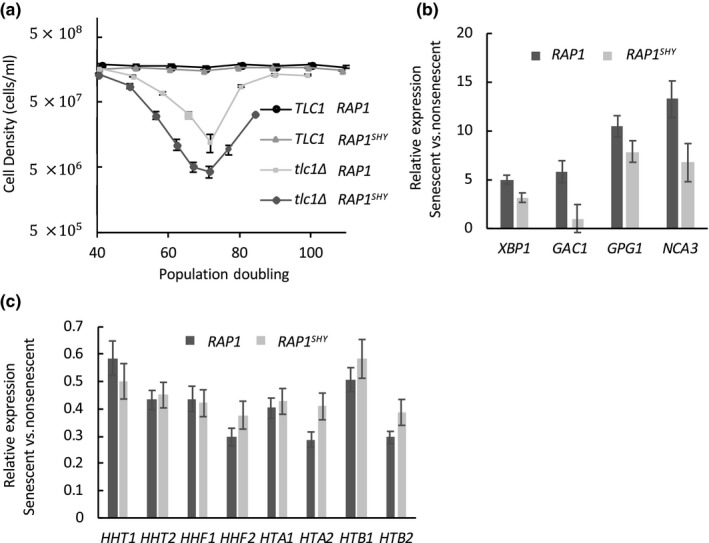
Rap1^SHY^ prevents upregulation of activated NRTS at senescence without affecting histone gene expression and the rate of senescence. (a) Rap1^SHY^ does not alter the rate of senescence. Senescence assay of *RAP1* (*n* = 3), *RAP1^SHY^* (*n* = 3), *tlc1Δ RAP1* (*n* = 7), *tlc1Δ RAP1^SHY^* (*n* = 7) spore products. (b) Rap1^SHY^ confers less NRTS activation at senescence. Relative expression of activated NRTS, measured by qPCR and normalized to nonsenescent strains (*p* < .05). (c) Histone gene repression is not affected by Rap1^SHY^. Relative histone gene expression was measured by qPCR and normalized to nonsenescent strains. All error bars indicate the standard error of the mean

We passaged both *tlc1Δ* and Rap1^SHY^ *tlc1Δ* cells to senescence by measuring the daily growth of liquid cultures seeded at a fixed starting concentration with cells obtained from the previous day of growth (see Methods). Taking senescence as the nadir of the growth curve before survivor formation, Rap1^SHY^ had no effect on the rate of senescence compared to WT Rap1 (Figure 4a). However, given the reduced NRTS activation observed when Rap1^SHY^ is overexpressed, we predicted that a similarly blunted NRTS profile would also be seen in Rap1^SHY^ at senescence. Indeed, this was confirmed by comparing relative mRNA expression in senescent and proliferating cells (Figure 4b). Interestingly, this suggests that the tested gene expression changes do not correlate with the rate of senescence. Previously, we have reported that Rap1 relocalization at senescence represses histone gene expression, and that artificial overexpression of all core histones will delay the rate of senescence (Platt et al., 2013), suggesting that the rate of senescence may be related to global histone levels. Consistent with this, the degree to which expression of all eight core histone genes was repressed at senescence was similar for cells expressing Rap1^SHY^ versus normal Rap1 (Figure 4c).

### 2.5 Asf1 contributes to Rap1‐dependent NRTS activation and histone displacement

**Figure 5:**
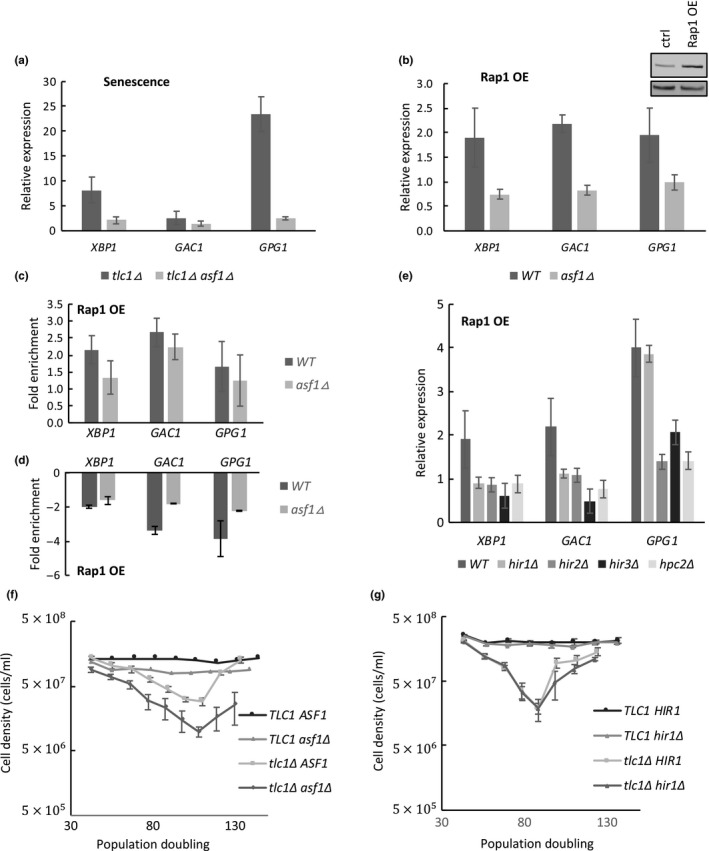
Asf1 is required for NRTS activation and histone displacement. (a) NRTS mRNA levels at senescence, measured by qPCR, and normalized to *ACT1* and nonsenescent strains. *asf1Δ tlc1Δ* double mutants have reduced NRTS activation compared to *tlc1Δ* strains (*N* = 5, *p* < .025). (b) Asf1 is required for NRTS activation in response to *NOP1‐*driven Rap1 overexpression (Rap1 OE) (*p* < .02). (c) ChIP‐qPCR of Rap1 in WT and *asf1Δ* strains with Rap1 OE driven by *GAL1*. Rap1 localization to promoters of activated NRTS is not affected by *ASF1* deletion (*p* values insignificant). ChIP signals are normalized to noninduced cells. (d) ChIP‐qPCR of histone H3 in WT and *asf1Δ* strains with Rap1 OE driven by *GAL1*. Histone displacement is diminished in *asf1Δ *strains (*p* < .05). ChIP signals are normalized to noninduced cells. (e) NRTS activation by Rap1 OE is blunted upon deletion of members of the HIR complex (*p* < .02). (f) *asf1Δ* does not affect the rate of senescence. Senescence assay with WT (*n* = 2), *asf1Δ* (*n* = 2), *tlc1Δ* (*n* = 5), and *tlc1Δ asf1Δ (n = 5*). (g) *hir1Δ* does not affect the rate of senescence. Senescence assay with WT (*n* = 2), *hir1Δ* (*n* = 2), *tlc1Δ* (*n* = 5), and *tlc1Δ hir1Δ (n = 5*). All error bars indicate the standard error of mean

Given the interaction of Rap1 with H3/H4 histone tetramers, we reasoned that H3/H4 histone chaperones might cooperate with Rap1 to displace nucleosomes at senescence. We tested the H3/H4 histone chaperone Asf1 because it is involved in both nucleosome assembly and disassembly during replication, transcription, and DNA repair, and because it is upregulated in senescent cells (Adkins, Howar, & Tyler, 2004; Nautiyal, DeRisi, & Blackburn, 2002; Zabaronick & Tyler, 2005). Deletion of *ASF1* results in histone gene misregulation (Sutton, Bucaria, Osley, & Sternglanz, 2001; Zabaronick & Tyler, 2005) and genome‐wide transcriptional changes, but does not affect the global level nor the stability of histone proteins (Gunjan & Verreault, 2003). We found that although *asf1Δ tlc1Δ* mutants did not senesce at a rate significantly different from *tlc1Δ* (Figure 5f), deletion of Asf1 significantly blunts the upregulation of activated NRTS at senescence (Figure 5a). To test if this reduced NRTS activation is related to regulation by Rap1, we tested the Asf1‐dependence of NRTS activation when Rap1 was overexpressed in wild‐type cells. Similar to its effects at senescence, deletion of *ASF1* reduced Rap1‐driven NRTS activation (Figure 5b) and displacement of nucleosomes from NRTS promoters (Figure 5d). However, Rap1 localization to the promoters did not depend on Asf1 (Figure 5c), indicating that facilitation of histone displacement by Asf1 occurs after the binding of Rap1 to promoters, apparently similar to the role of the Rap1 SHY patch described above.

Asf1 partners with different complexes to chaperone histones in different contexts. Asf1 interacts with the HIR complex, comprising Hir1, Hir2, Hir3, and Hpc2 proteins, to regulate nucleosome assembly and disassembly during transcription or DNA repair. In contrast, during DNA replication, Asf1 interacts with the second subunit of the CAF‐I complex, comprising Cac1, Cac2, and Cac3, to promote nucleosome assembly. Deletion of any member of the HIR complex blunted NRTS activation caused by overexpressed Rap1 (Figure 5e), but deletion of *cac1Δ*, *cac2Δ,* and *cac3 Δ* had no effect (Figure S5). This is consistent with the fact that cells arrest in G2/M at senescence and therefore would not be expected to utilize the replication‐dependent pathway. Furthermore, similar to *ASF1* deletion, *HIR1* deletion did not affect the rate of senescence of *tlc1Δ* cells (Figure 5g). Therefore, deletion of *ASF1* or *HIR1*, or mutation of the Rap1 SHY patch, each prevent normal NRTS upregulation by Rap1 without impacting the rate of senescence, suggesting that upregulation of NRTS, at least those tested, are not main drivers of this rate.

## 3 DISCUSSION

### 3.1 Direct Rap1‐histone interactions are involved in Rap1‐mediated chromatin opening

There is long‐standing evidence that Rap1 can bind to nucleosomal DNA both in vivo and in vitro (Koerber et al., 2009; Lickwar et al., 2012; Rossetti et al., 2001). Notably, single‐nucleotide resolution ChIP‐exo shows that not only does histone occupancy not interfere with Rap1 binding, but that high‐affinity Rap1 binding sites and Rap1 occupancy are in fact more common in nucleosomal than nonnucleosomal regions of the genome (Rhee & Pugh, 2011). Rap1, nonetheless, encourages nucleosome displacement, as Rap1‐bound regions have generally low nucleosome occupancy that depends on the binding of Rap1 (Ganapathi et al., 2011; Lieb, Liu, Botstein, & Brown, 2001; Platt et al., 2013; Rhee & Pugh, 2011; Yarragudi et al., 2004). The fact that telomeric chromatin, which includes one Rap1 monomer bound to approximately every 18 bp of the telomere repeat sequence, is largely nucleosome‐free provides another apparent example of the nucleosome‐displacing activity of Rap1 (Gilson, Roberge, Giraldo, Rhodes, & Gasser, 1993; Williams, Levy, Maki‐Yonekura, Yonekura, & Blackburn, 2010; Wright, Gottschling, & Zakian, 1992).

Here we report that in addition to high‐affinity Rap1‐DNA interactions, Rap1 can also interact directly with H3/H4 histone tetramers. The SANT domain is necessary for such binding, and is facilitated by amino acids 392–394 (SHY) within the domain, although other points of contact apparently also exist, including within the C‐terminal region. Mutation of amino acids SHY to AAA results in deficiencies in Rap1‐histone interactions in vitro and in vivo, as well as blunted histone displacement and gene activation. These findings are consistent with previous mapping studies demonstrating that the N‐terminal and C‐terminal regions of Rap1 are dispensable for interaction with nucleosomal binding sites (Rossetti et al., 2001) and chromatin opening (Yu et al., 2001).

The SHY patch is located within the turn immediately C‐terminal to the second helix in the three‐helix bundle of the SANT domain. Crystal structures of Rap1 bound to DNA (Konig et al., 1996; Matot et al., 2012) show that helices 2 and 3 form a helix‐turn‐helix motif that docks deep in the major groove of DNA, and that the side chains of the SHY patch point away from the Rap1‐DNA interaction surface. Of note, the C‐terminal region of the second Myb domain (amino acids 592–601) forms a long loop which wraps around the DNA helix, contacts both DNA strands, and ends with a “clamp” formed though interaction with SANT domain residues that partially overlap the SHY patch. Specifically, Y592 interacts with G400 and Q401, whereas K597 interacts with S392 and P396. Mutation of Y592 and K597 in the full‐length protein results in no changes in the migration profile of DNA–protein complexes visualized by EMSA, though DNA binding affinity measured by ITC is reduced by a factor of two (Matot et al., 2012). Similarly, no changes in EMSA profiles for telomeres, natural Rap1 binding sites, or representative NRTS promoters were observed with S392 mutation, though its ITC profile has not been tested directly. However, our ChIP‐qPCR measurements at NRTS promoters reveal no apparent decrease in Rap1^SHY^ occupancy, and so the reduced ability of Rap1^SHY^ to displace nucleosomes and activate transcription is apparently not a consequence of reduced levels of Rap1 at promoters (Figure 3b). Whether interference with the “clamp” per se contributes to the compromised nucleosome displacement by Rap1^SHY^ will require additional studies.

The fact that direct Rap1‐histone interactions contribute to nucleosome displacement might seem counterintuitive at face value, as a simple energetic consequence of histone binding by Rap1 bound to a DNA target site should be to tether a nucleosome to the site. However, it is noteworthy that Rap1 interacts specifically with the H3/H4 histone tetramers, and not the H2A/H2B histone dimers. Given the sequential assembly and disassembly of nucleosomes—H3/H4 at the core and H2A/H2B on the periphery—this suggests that Rap1 may interact preferentially with a partially disassembled nucleosome. Nucleosomes naturally undergo transient unwrapping under physiological conditions, resulting in DNA partially wrapped around a hexasome or tetrasome through loss of one or both of H2A/H2B dimers (Chen et al., 2017; Li & Widom, 2004). Such “breathing” not only allows exposure of DNA sequences for transcription factor binding, but also bares the tetrameric core for protein–protein interactions. Therefore, Rap1 binding to H3/H4 tetramers may drive the dynamic equilibrium of wrapped and partially unwrapped nucleosomes toward the unwrapped state, possibly by preventing H2A/H2B reassembly. Alternatively, or in addition, it may also alter the conformation of the tetrasome in a fashion that facilitates full nucleosome disassembly by H3/H4 histone chaperones such as Asf1. This is similar to the functions of some histone PTMs such as H3K56ac, which destabilize nucleosomes and enable them to be more easily disassembled (Williams, Truong, & Tyler, 2008).

Rap1 is functionally similar to pTFs in eukaryotes. pTFs are the first to bind to target sites in compact chromatin and initiate the sequential binding of other factors, possibly through opening up local chromatin. Well‐known pTFs such as FoxA have DNA binding domains consisting of helix‐turn‐helix motifs flanked by “wings” of polypeptides, allowing the motif to bind alongside one side of DNA without interfering with the binding of histones on the other side (Soufi et al., 2015;Zaret & Carroll, 2011). Such a DBD secondary structure and its orientation on DNA are very similar to those observed in Rap1. In addition, FoxA has a C‐terminal domain that interacts directly with core histones H3 and H4 (Cirillo et al., 2002). Interestingly, while the C‐terminal region of Rap1 also contributes to histone binding abilities, the Rap1 DBD alone is able to open local chromatin (Yu et al., 2001), consistent with our observations that direct histone interactions in the SANT domain, via amino acids SHY, are important for Rap1’s functions as a pTF.

### 3.2 Gene expression changes can be uncoupled from the rate of senescence

Previously, we reported several functions of Rap1 at senescence: first, it represses histone gene expression and contributes to global downregulation of histones; second, it contributes to local nucleosome losses at promoters of upregulated NRTS (Platt et al., 2013); third, Rap1 drives the rate of senescence, probably through regulation of histone dynamics, as diminishment of Rap1 levels via destabilization of the Rap1 mRNA (DAmP allele) or artificial overexpression of core histones can delay the rate of senescence.

Here, we explore mechanisms of Rap1‐mediated local nucleosome losses. We characterize an amino acid patch—residues SHY in the SANT domain—that contains one or more residues required for direct binding of Rap1 to histones. Mutation of SHY results in deficient nucleosome clearance at NRTS promoters and subsequently reduced activation of NRTS. However, *RAP1^SHY^* affects neither the rate of senescence nor histone gene repression. This is consistent with our previous finding that histone gene repression at senescence does not involve nucleosome losses from the histone gene promoters (Platt et al., 2013).

Similarly, we found Rap1‐mediated gene expression changes at senescence require the histone H3/H4 chaperone Asf1 and the HIR complex. Deletion of *ASF1* results in blunted NRTS upregulation at senescence. Similar loss of activation was seen when *ASF1* or genes encoding members of the HIR complex were deleted under settings of Rap1 overexpression, which we previously found was sufficient for selective NRTS upregulation. However, much like the *RAP1^SHY^* mutation, despite causing substantial losses in NRTS activation, deletion of *ASF1* or *HIR1* also do not affect the rate of senescence.

This begs the question of how gene expression changes—in particular, wide‐spread gene upregulation that has been observed in multiple senescent models—relate to the rate of senescence. As has been reported extensively, senescent cells are accompanied by changes in chromatin organization and gene expression (De Cecco et al., 2013; Feser & Tyler, 2011; Lackner, Hayashi, Cesare, & Karlseder, 2014; Sedivy, Banumathy, & Adams, 2008; Shah et al., 2013). Given the large numbers of genes affected at senescence, it has been difficult previously to study the relationship between gene expression changes and the rate of senescence without perturbing fundamental cellular processes. Here, by exploring the mechanisms of Rap1‐mediated gene expression changes at senescence, we were able to generate a separation‐of‐function Rap1 mutant that affected in particular Rap1’s functions at activated NRTS. However, despite diminished NRTS gene activation, the rate of senescence was not changed, showing that gene expression can be uncoupled from the rate of senescence. This may also be true for mammalian factors controlling gene expression changes in senescent cells, because several can be manipulated to blunt the altered expression without bringing senescent cells out of cell cycle arrest (Correia‐Melo et al., 2016; Georgilis et al., 2018; Nacarelli et al., 2019; Tasdemir et al., 2016). This is encouraging, as it indicates that negative aspects of cell senescence can be blocked without compromising its tumor‐suppressive properties.

## 4 EXPERIMENTAL PROCEDURES

### 4.1 Yeast strains and plasmids

All experiments are performed using BY4741/4742 background, and deletion strains are from the haploid yeast knockout collection or were constructed using standard gene replacement techniques. Plasmids were made using Gateway cloning methods. Site‐directed mutagenesis to generate Rap1 *Escherichia coli* expression plasmids with AAA mutations in the SANT domain was performed using QuickChange primer design (Agilent) and primer extension using Phusion HF to introduce the changes into pGST‐SANT‐6xHis (BSS48); all mutations were verified by sequencing. All strains and plasmids used are listed in Table S1, and primers used for mutagenesis are listed in Table S2.

### 4.2 Expression and purification of Rap1 and Rap1 derivatives

All Rap1 proteins were N‐terminally tagged with GST, and some were also C‐terminally tagged with a 6x‐His tag, as indicated in the text.

For Ni‐NTA purifications, 200 ml of BL21(DE3) cells containing expression plasmids GST‐Rap1‐6X‐His or GST‐SANT‐6X‐His were grown at 37°C to a cell concentration of OD (600) 0.4, and then induced with 1 mM IPTG overnight at room temperature. Protein was purified according to Ni‐NTA Purification System protocol by Novex with the following modifications. Cell pellets were resuspended in 20 ml of native binding buffer (50 mM NaH_2_PO_4_, pH 8.0, 500 mM NaCl, 1% Triton X‐100, 1 mg/ml lysozyme, benzonase 1,000 Units). Protein lysate, treated with benzonase and clarified by centrifugation as recommended, was loaded onto Ni‐NTA resin. After incubation and washing, to ensure removal of any contamination by residual DNA, an additional on‐column benzonase digestion was performed (10 mM Tris‐Cl, pH 8.0, 100 mM NaCl, 1 mM DTT, 1.5 mM MgCl_2_, benzonase 500 Units) at room temperature for 15 min. High‐sensitivity measurements of DNA by Qubit showed minimal amounts of DNA (40 Rap1 molecules for every base pair of DNA). Protein was eluted using 10 ml elution buffer (50 mM NaH_2_PO_4_, pH 8.0, 500 mM NaCl, 250 mM imidazole). Protein of interest was quantified using SDS‐PAGE gel electrophoresis and BSA standards and then flash‐frozen for storage.

For GST‐only protein purifications, 200 ml of BL21(DE3) cells containing the various Rap1 expression plasmids were grown and induced as described above. Cell pellets were flash‐frozen and stored at −80°C. The GST‐Rap1 was purified using Methods described in Schäfer, Seip, Maertens, Block, and Kubicek (2015) with modifications. Clarified protein lysate treated with benzonase was loaded onto 2.5 ml of glutathione resin (GE Healthcare). An on‐column benzonase digestion as described above was performed to remove residual DNA. Protein concentration was determined by boiling 10 µl of resin in 2× SDS‐PAGE buffer and SDS‐PAGE gel electrophoresis using BSA as standard.

### 4.3 GST histone pull‐down assay

Protein purified by Ni‐NTA were thawed and diluted using 1 volume of binding buffer (50 mM Tris‐Cl, pH 7.5, 1 mM BME, and 150–750 mM NaCl, as indicated in text), and incubated overnight with equilibrated glutathione resin (50 µl). GST‐6X‐His proteins were used as negative controls. After supernatant is removed, resin was washed twice in 500 µl of binding buffer. Proteins purified using GST protein purifications were used directly. Histones were purified as described in Ricketts et al. (2015). The pull‐down assay was performed by incubating 2 µM purified histones and desired concentration of GST‐tagged protein for 90 min in 500 µl of binding buffer with rotation at 4°C. Resin was then washed 4× with 500 µl of binding buffer before elution of proteins by boiling in 2× SDS‐PAGE buffer. 10% of the pull‐down were then analyzed by SDS‐PAGE and Coomassie staining using Coomassie Brilliant Blue G‐250 or by Western blot (anti‐H3, Abcam ab 1791, 1:2,000).

### 4.4 Coimmunoprecipitation from whole‐cell extracts

BY4741 cells containing *NOP1‐*driven HA‐tagged Rap1 or Rap1^SHY^ expression plasmids were grown in SC‐His to a concentration of 2 × 10^7^ cells/ml. Cells were suspended in lysis buffer (50 mM Hepes‐KOH, pH 8, 5% glycerol, 300 mM KCl, 0.1% NP‐40, 0.1 mM DTT, 1× EDTA‐free protease inhibitor cocktail (Roche), 1 mM PMSF, 1 µg/ml pepstatin, 1 µg/ml leupeptin, 1 µg/ml aprotinin, 5 mM NaF), and subjected to mechanical disruption with bead beating at 4°C (60 s, 4×). After removal of beads, MgCl_2_ and benzonase were added to a final concentration of 2 mM and 25 U/ml, respectively, and the WCE was incubated while rotating at room temperature for 30 min, followed by addition of 4 mM of EDTA to quench benzonase digestion. Protein concentration in the clarified WCE was determined by Bradford quantitation, and 500 µg of protein in 150 µl of lysis buffer was used for each coIP. WCE were diluted in equal volume of 50 mM Tris, 5% glycerol, 1 mM EDTA containing 2× protease inhibitors and incubated with HA‐antibody (Abcam ab 9110) bound Dynabeads for 2 hr at 4°C. Beads were then washed in lysis buffer, and bound proteins were solubilized with 2× SDS sample buffer and analyzed by standard SDS‐PAGE and Western blotting.

### 4.5 Chromatin immunoprecipitation

BY4741 cells containing *GAL1‐*driven expression plasmids were grown in SC‐Ura + raffinose to a concentration of 0.5 × 10^7^ cells/ml, then induced with galactose (final concentration 2%) for 130 min. Cells were cross‐linked with 1% formaldehyde solution (w/v), methanol free (Thermo Scientific Ref 28908), for 30 min at room temperature and quenched with 125 mM (final concentration) glycine. Cell pellets were frozen and stored at −80°C. For ChIP, cells were lysed in FA lysis buffer (50 mM Hepes‐KOH, pH 7.5, 140 mM NaCl, 1 mM EDTA, 0.1% Triton, 1 mM PMSF, 2 µg/ml aprotinin, 2 µg/ml leupeptin, 2 µg/ml pepstatin A, 1× protease inhibitor cocktail) and subjected to mechanical disruption with bead beating (60 s, 6×). Lysate was sonicated with Covaris S220, peak power 240.0, duty factor 20.0, cycles/burst 200, time: 420 s, and the protein concentration in the WCE was determined using Bradford quantitation. About 1.5 mg of WCE was used for pull‐down of HA‐tagged proteins, and 0.5 mg of WCE was used for H3 pull‐down. 200 µl of Protein G Dynabeads (Invitrogen) was blocked with Block Solution (0.5% BSA) and incubated with the appropriate amount of antibodies (anti‐HA, Abcam ab 9110, 7.5 µg, anti‐H3 Abcam ab 1791, 5 µg, and rabbit IgG, ImmunoPure 31207, 7.5 and 5 µg, respectively) in 500 µl of Block Solution for a minimum of 6 hr. WCE were incubated with respective antibodies or IgG overnight at 4°C with rotation. Beads were washed 2× with FA lysis buffer, 1× with FA lysis buffer/500 mM NaCl, 2× with LiCl solution (10 mM Tris‐Cl, pH 8.0, 0.25 M LiCl, 1 mM EDTA, 0.5% NP‐40), 2× with TE + 0.1% NP‐40, and then eluted with TES (50 mM Tri‐Cl, pH 8.0, 10 mM EDTA, 1% SDS) three times for 15 min at 65°C. Eluted DNA was reverse cross‐linked with 200 mM NaCl at 65°C overnight and subjected to 1 hr each of RNase A (0.4 mg/mg) and proteinase K (0.3 mg/ml) incubations at 37°C and purified using QIAgen MinElute Spin Columns. qPCR was performed as described in Platt et al. (2013). Statistical analyses were performed using two‐tailed unpaired *t*‐tests.

### 4.6 Quantitation of mRNA analysis

mRNA expressions were quantified using methods described in Platt et al. (2013). All mRNA analyses for Rap1 or mutant overexpression were performed using protein expression driven by the *NOP1* promoter. Signals were calculated using standard curves of pooled cDNA samples and normalized to *ACT1*. Error bars indicate standard error of mean. P‐values were calculated using two‐tailed unpaired *t*‐tests.

### 4.7 Electrophoretic mobility shift assays

Proteins were purified using Ni‐NTA and GST resin and quantified using Coomassie blue staining with BSA standards. DNA probes were generated using polynucleotide kinase to ^32^P‐end label oligos, followed by annealing to their unlabeled complementary strands (Table S6). The fraction of active protein was similar for Rap1 and Rap1^SHY^ preparations (~85%), and was quantified by incubating 10 nM protein as measured by Coomassie blue staining with increasing amounts of *TEF2* probe (0–100 nM), taking the fraction of protein–DNA complex formation at saturated DNA concentrations as a measure of active protein. For EMSAs, 0.5 nM ^32^P‐labeled duplexes were incubated with increasing concentrations of active protein in binding buffer (20 mM Hepes‐KOH, pH 8, 100 mM KCl, 10 µg/ml BSA, 1 mM EDTA, 2 mM MgCl_2_, 5% glycerol) for 30 min at room temperature. Reactions were loaded on 6% DNA retardation gel (Invitrogen) and electrophoresis was conducted at 100V at 4°C. Radioactive signals were visualized using Typhoon FLA 7000.

### 4.8 Integration of SHY to AAA mutation in the *RAP1* locus

Genome editing was performed using the 50:50 method for PCR‐based seamless genome editing in yeast (Horecka & Davis, 2014). Forward and reverse primers encompassing SHY→AAA mutation and homologous to *URA3* were used for amplification of *URA3* from pRS306 by PCR. As *RAP1* is an essential gene in *S. cerevisiae*, the mutation was introduced into diploid cells (*TLC1/tlc1Δ*), which were then sporulated and dissected. Haploids containing the mutation were confirmed via sequencing.

### 4.9 Southern blotting

Telomere lengths were determined as described (Johnson et al., 2001), using XhoI digested DNA run on a 1% agarose gel, transferred to a Hybond‐XL membrane, and probed using a radio‐labeled telomere Y’ fragment.

### 4.10 Senescence assays

Senescence assays were performed as described in Platt et al. (2013). In short, cells from the Yeast Knockout Library were mated with early generation *tlc1*Δ*::LEU2* and the diploids were grown for 60 doublings to allow for equilibration of telomere lengths. Diploids were sporulated, dissected, and genotyped. All comparisons between different genotypes were derived from the same tetrad heterozygous from *tlc1*Δ deletion and other mutations of interest (e.g., *RAP1/RAP1^SHY^*) to ensure inheritance of similar telomere length. Spore products were grown in YPAD liquid media and passaged every 22 hr. For each passage, cells were counted using a Coulter counter and diluted to 10^6^ cells/ml in 5 ml of liquid media. Cell counts were used to determine population doublings (PD), and the point of senescence was determined from the PD displaying the lowest level of growth. Cells for mRNA expression at senescence were obtained ~5 PDs prior to the nadir to avoid formation of survivors, and grown 2–3 more doublings to a density of 1 × 10^7^ cells/ml in fresh medium before harvest.
