# Supplementary material for: Rap1‐mediated nucleosome displacement can regulate gene expression in senescent cells without impacting the pace of senescence
Source: Aging Cell. 2019 Nov 19;19(1):e13061. doi: 10.1111/acel.13061 (PMC6974733; doi:10.1111/acel.13061)
Supplement: Supplementary file 1 [file ACEL-19-e13061-s001.pdf]

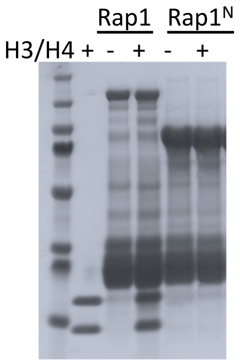

Fig. S1. The Rap1 N terminal fragment does not bind H3/H4 . GST pull-down histone binding assay with full-length Rap1 and Rap1^{N} (400 mM NaCl). Full-length Rap1 (2 μM) binds H3/H4 tetramers (2 μM) at equal-molar concentrations, but Rap1N does not bind H3/H4 even when in vast excess (15 μM).

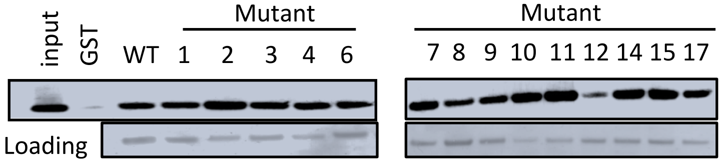

Fig. S2. **Triple alanine screen .** Immunoblot analysis of GST pull-down of histones. Pull-down was performed with equimolar GST-SANT (0.5 μM) and histones 0.5 μM) at 400 mM NaCl. Bottom panel is the blot stained with Ponceau S as a loading control. Mutants 5 and 13 did not express well in *E. coli* and so were not tested.

A.

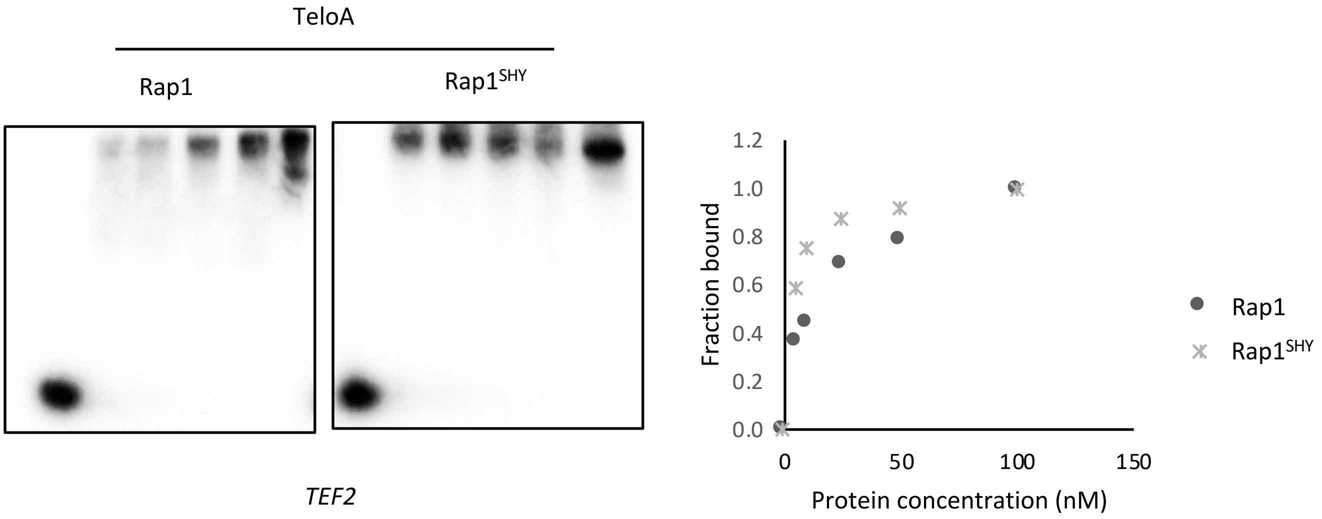
TeloA
Rap1
Rap1SHY
Fraction bound
1.2
1.0
0.8
0.6
0.4
0.2
0.0
0
50
100
150
Protein concentration (nM)
Rap1
Rap1SHY

B.

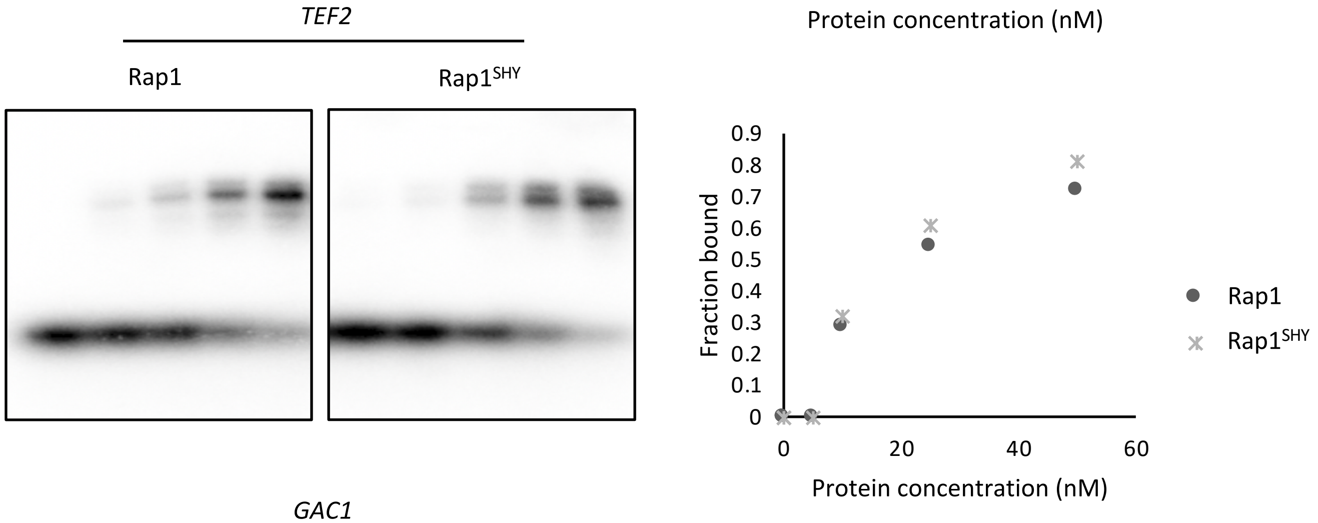
TEF2
Rap1
Rap1SHY
Fraction bound
0.9
0.8
0.7
0.6
0.5
0.4
0.3
0.2
0.1
0
0
20
40
60
Protein concentration (nM)
Rap1
Rap1SHY

C.

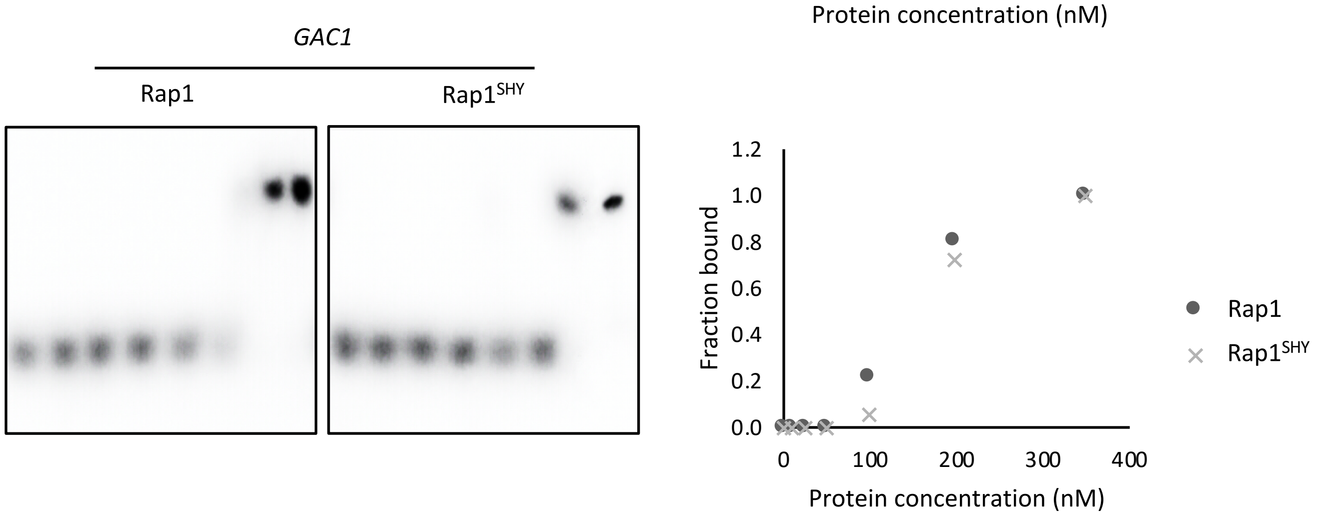
GAC1
Rap1
Rap1SHY
Fraction bound
1.2
1.0
0.8
0.6
0.4
0.2
0.0
0
100
200
300
400
Protein concentration (nM)
Rap1
Rap1SHY

D.

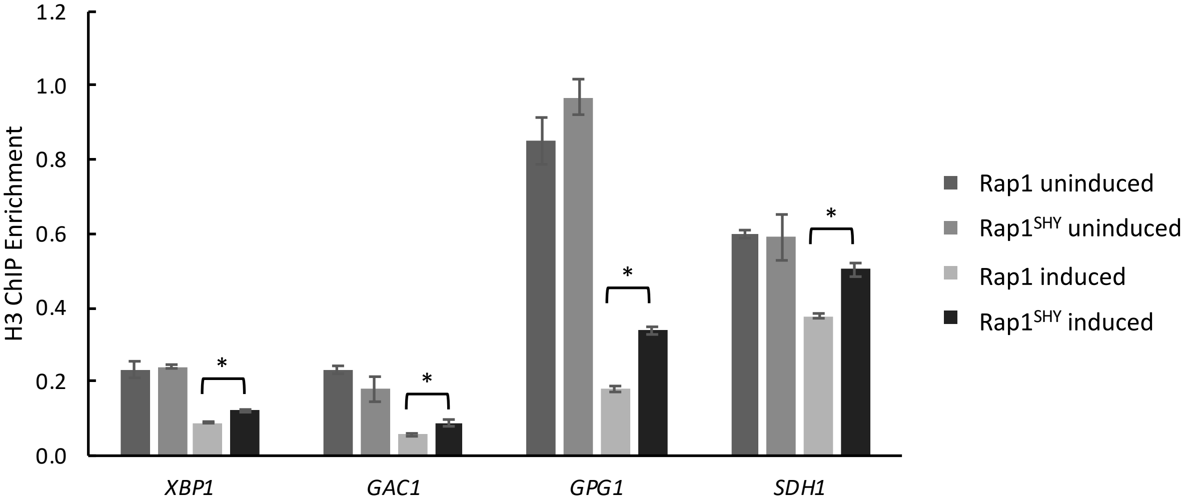

E.

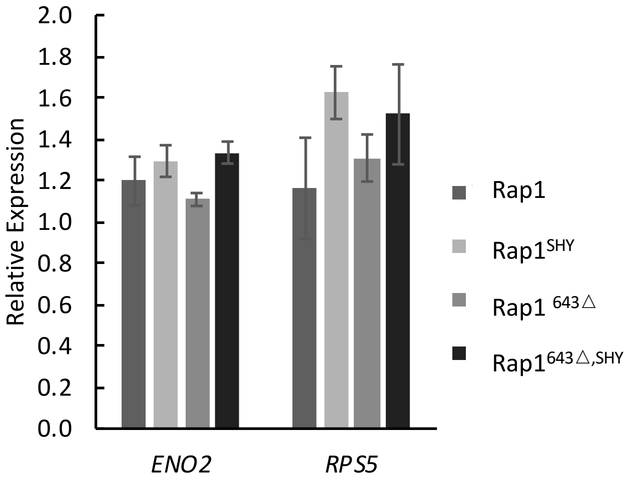

F.

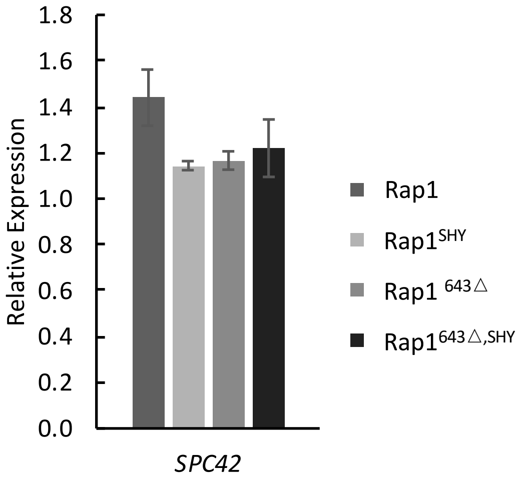

Fig. S3 The SHY mutation does not compromise Rap1 binding to natural and NRTS target DNA, and it does not affect expression of tested natural Rap1 target and non-target genes. A. EMSA of Rap1 and Rap1^{SHY} binding to telomeric sequence TeloA. Proteins were titrated from 0.5 nM to 100 nM. Quantifications were performed by measuring the fraction of probe bound to protein at a given protein concentration. Rap1^{SHY} exhibited higher affinity for the telomeric sequence compared to Rap1. **B.** EMSA of Rap1 and Rap1^{SHY} binding to the *TEF2* promoter sequence, a canonical Rap1 binding site. Proteins were titrated from 5 nM to 50 nM. Rap1 and Rap1^{SHY} showed similar binding affinities. **C.** EMSA of Rap1 and Rap1^{SHY} binding to a NRTS promoter sequence of *GAC1*. Proteins were titrated from 5-500 nM. Rap1 and Rap1^{SHY} displayed similar affinities, and has a Kd approximately 10-fold lower than that observed in B. **D.** H3 levels at the promoters of the upregulated NRTS in induced and uninduced cells, normalized to *MDP1*. No significant differences were observed in uninduced Rap1 and Rap1^{SHY} strains, but Rap1^{SHY} had less nucleosome displacement compared to WT after induction (*$p<0.05$). **E-F.** mRNA levels of glycolytic gene *ENO2*, ribosomal gene *RPS5*, and non-Rap1 target gene *SPC42* in cells overexpressing the indicated Rap1 proteins, measured by qPCR and normalized to vector control and *ACT1*. Rap1^{SHY} and Rap1$^{643\Delta, SHY}$ do not significantly compromise expression of natural Rap1 targets or non-Rap1 target genes.

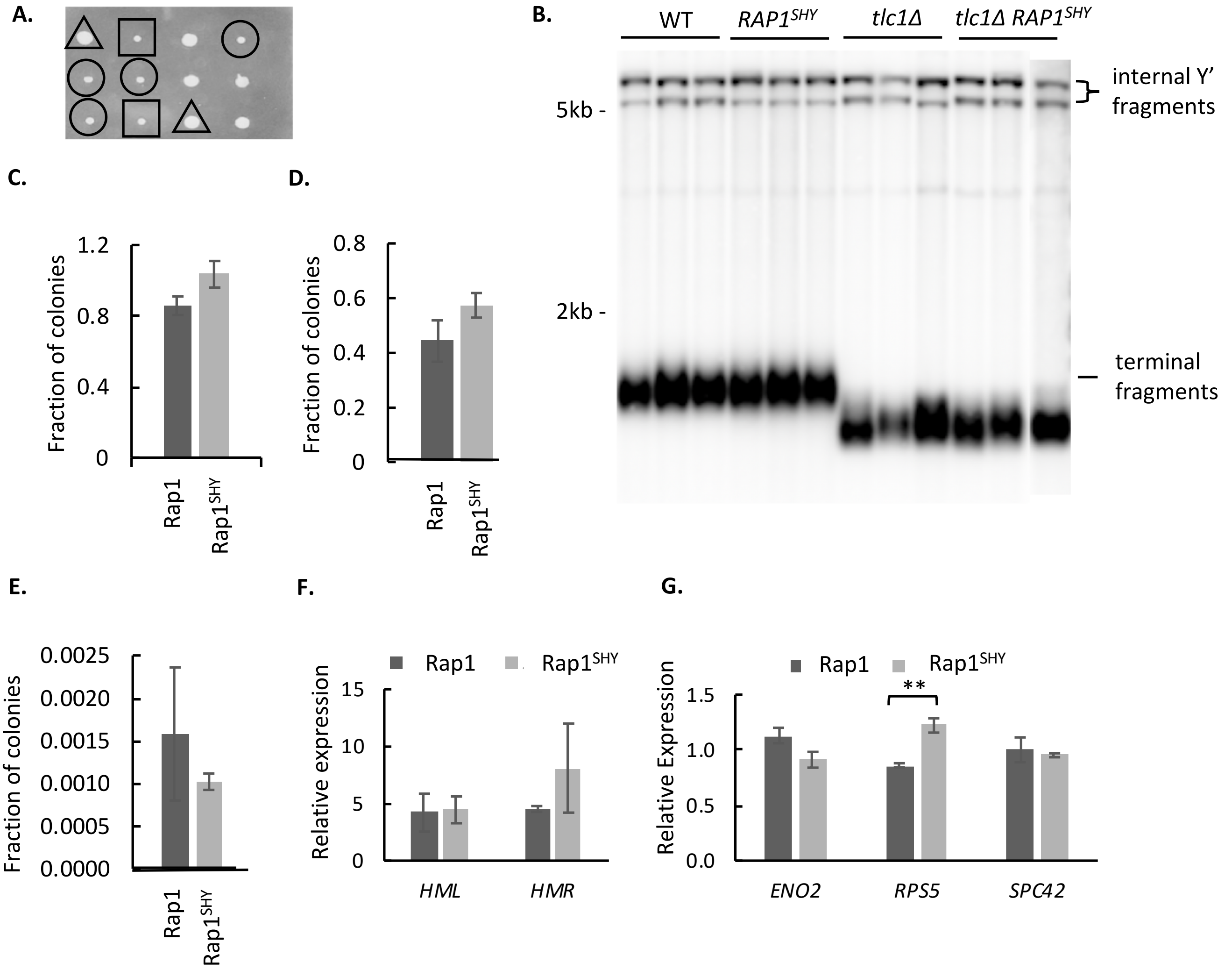

Fig. S4 Characterization of $Rap1^{SHY}$. A. *RAP1* and $RAP1^{SHY}$ haploid spore products from tetrad dissections of sporulated $RAP1^{+/SHY}$ *TLC1/tlc1△* diploids. $RAP1^{SHY}$ in the endogenous locus has a slower growth phenotype. However, $RAP1^{SHY}$ TLC1 (square) does not have a significant growth difference from $RAP1^{SHY}$ *tlc1△* (circle). Triangles indicate *RAP1 tlc1△*, and unmarked colonies are *RAP1 TLC1*. **B.** Southern blot of telomere lengths using the ^{32}P-labeled Y' telomeric probe. No significant differences were observed between Rap1 and $Rap1^{SHY}$ strains or in their respective *tlc1Δ* strains (50 PDs after spore germination). Note that an inadvertently unloaded lane between the last two lanes was cropped from the figure. **C.** Colony-forming efficiency. 200 *RAP1* and $RAP1^{SHY}$ cells were plated and colonies counted. $RAP1^{SHY}$ forms a similar percentage of colonies compared to WT. **D-E.** Telomeric silencing. Cells bearing the *URA3* marker in the VII-L telomere were pre-grown in either complete medium (D) or SC-Ura (E). 200 (D) and 400,000 (E) cells were plated on 5-FOA plates to select for silencing of the *URA3* gene. $RAP1^{SHY}$ had no defects in telomere silencing compared to WT. **F.** mRNA expression of the silent mating type normalized to *ACT1*. mRNA was harvested from *RAP1* and $RAP1^{SHY}$ strains and no differences in *HML* and *HMR* expression were observed by qPCR. **G.** mRNA expression measured by qPCR of natural Rap1 targets glycolytic gene *ENO2*, ribosomal gene *RPS5*, and non-Rap1 target *SPC42*, normalized to *ACT1*. $RAP1^{SHY}$ has higher glycolytic gene expression compared to *RAP1*. All error bars are standard error of the mean (**$p<0.01$, N=3).

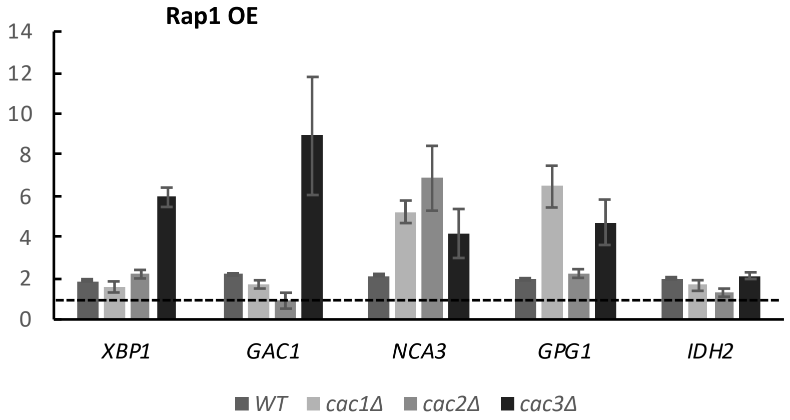

Fig. S5. The CAF complex does not contribute to Rap1-mediated NRTS activation. mRNA levels of activated NRTS, measured by qPCR and normalized to *ACT1* and vector control. Rap1 overexpression is driven by *NOP1*.
