# Supplementary material for: Rap1‐mediated nucleosome displacement can regulate gene expression in senescent cells without impacting the pace of senescence
Source: Aging Cell. 2019 Nov 19;19(1):e13061. doi: 10.1111/acel.13061 (PMC6974733; doi:10.1111/acel.13061)
Supplement: Supplementary file 2 [file ACEL-19-e13061-s002.docx]

**Supplementary Table 1. Yeast strains**

| **Strain** | **Genotype** | **Reference (source)** |
| --- | --- | --- |
| BY4741 | MATa *his3Δ1 leu2Δ0 met15Δ0 ura3Δ0* | Brachmann CB, et al. (1998) |
| BY4742 | MATα his3Δ1 leu2Δ0 lys2Δ0 ura3Δ0 | Brachmann CB, et al. (1998) |
| *ASF1/asf1Δ* TLC1/tlc1Δ* | *BY4741/BY4742 ASF1/asf1Δ::KanMX, TLC1/tlc1Δ::LEU2* | This study |
| *HIR1/hir1Δ TLC1/tlc1Δ* | *BY4741/BY4742 HIR1/hirΔ::KanMX, TLC1/tlc1Δ::LEU2* | This study |
| *HIR2/hir2Δ TLC1/tlc1Δ* | *BY4741/BY4742 HIR2/hir2Δ::KanMX, TLC1/tlc1Δ::LEU2* | This study |
| *HIR3/hir3Δ TLC1/tlc1Δ* | *BY4741/BY4742 HIR3/hir3Δ::KanMX, TLC1/tlc1Δ::LEU2* | This study |
| *HPC2/hpc2Δ TLC1/tlc1Δ* | *BY4741/BY4742 HPC2/hpc2Δ::KanMX, TLC1/tlc1Δ::LEU2* | This study |
| *CAC1/cac1Δ TLC1/tlc1Δ* | *BY4741/BY4742 CAC1/cac1Δ::KanMX, TLC1/tlc1Δ::LEU2* | This study |
| *CAC2/cac2Δ TLC1/tlc1Δ* | *BY4741/BY4742 CAC2/cac2Δ::KanMX, TLC1/tlc1Δ::LEU2* | This study |
| *CAC3/cac3Δ TLC1/tlc1Δ* | *BY4741/BY4742 CAC3/cac3Δ::KanMX, TLC1/tlc1Δ::LEU2* | This study |
| *SS234* | *BY4741/BY4742 RAP1/RAP1^SHY^, TLC1/tlc1Δ::LEU2* | This study |
| *SS236* | *BY4741 RAP1^SHY^* | This study |

*All KanMX-replaced alleles (in *ASF1, HIR1/2/3, HPC2,* and *CAC1/2/3*), are derived from the yeast haploid KO collection in BY4741

**Supplementary Table 2. Plasmids**

| **Plasmid** | **Genotype** | **Reference** |
| --- | --- | --- |
| BJP201 | pAG423-*NOP1*p::ccdb | Platt et al., 2013 |
| BJP69 | pAG423-*NOP1*p::*RAP1* | Platt et al., 2013 |
| BSS20 | pGST-His | This study; constructed using Gateway destination vector pDEST15 |
| BSS18 | pGST-*RAP1* | “ |
| BSS104 | pGST-*RAP1*-6xHis | “ |
| BSS48 | pGST-SANT-6xHis | “ |
| BSS54-BSS70 | pGST-SANT*-6x-His | “ |
| BSS122 | pGST-*RAP1*^N^ | “ |
| BSS124 | pGST-*RAP1*^C^ | “ |
| BSS143 | pGST-*RAP1*^CΔ^ | “ |
| BSS163 | pGST-*RAP1* ^CΔSHY^-6xHis | “ |
| BSS165 | pGST-*RAP1* ^643ΔSHY^-6xHis | “ |
| BSS167 | pGST-*RAP1* ^SHY^-6xHis | “ |
| BSS197 | pAG423-*NOP1*::HA-*RAP1* | This study; constructed BJP201 |
| BSS200 | pAG423-*NOP1*::HA-*RAP1*^SHY^ | This study; via site directed mutagenesis of BSS197 |
| BSS186 | pAG423-*NOP1*::HA-*RAP1*^643Δ^ | “ |
| BSS202 | pAG423-*NOP1*::HA-*RAP1*^643ΔSHY^ | This study; via site directed mutagenesis of BSS186 |
| BSS209 | pAG426-*GAL1*::HA-*RAP1* | This study; constructed using Gateway destination vector pAG426-GAL1 |
| BSS211 | pAG426-*GAL1*::HA-*RAP1*^SHY^ | This study; via site directed mutagenesis of BSS209 |

*Triple alanine screen: every three consecutive amino acids in the SANT domain (amino acids 360-410) was mutated to AAA

**Supplementary Table 3. Triple alanine mutation primers**

| Residues | Primer Sequence |
| --- | --- |
| 359-361 | a46g_a47c_a49g_a50c_t54a_  5'- catcttcctcatctgtaaaagatgctgcagcgtgggagggcaaagcgccagcc -3'  5'-ggctggcgctttgccctcccacgctgcagcatcttttacagatgaggaagatg-3' |
| 362-364 | t55g_t58g_t59c_a61g_  5'-aaaataaactcatcttcctcatctgcagcagcagctttattgtgggagggcaaagcg-3'  5'-cgctttgccctcccacaataaagctgctgctgcagatgaggaagatgagtttatttt-3' |
| 365-367 | \| a65c_a68c_a71c_  5'-tcccacaataaagcttcttttacagctgcggcagatgagtttattttggatgttgtg-3'  5'-cacaacatccaaaataaactcatctgccgcagctgtaaaagaagctttattgtggga-3' \| \| --- \| |
| 368-370 | a74c_a77c_t79g_t80c_  5'-gttggattttttctcacaacatccaaaatagccgcagcttcctcatctgtaaaagaagctttattgt-3'  5'-acaataaagcttcttttacagatgaggaagctgcggctattttggatgttgtgagaaaaaatccaac-3' |
| 371-373 | a82g_t83c_t85g_t86c_a89c_  5'-ggttggattttttctcacaacagccgcagcaaactcatcttcctcatctgtaaaagaagctttattgtgggag-3'  5'-ctcccacaataaagcttcttttacagatgaggaagatgagtttgctgcggctgttgtgagaaaaaatccaacc-3' |
| 374-376 | t92c_t95c_a97g_g98c_  5'-tgttgtacgcctggttggattttttgccgcagcatccaaaataaactcatcttcctc-3'  5'-gaggaagatgagtttattttggatgctgcggcaaaaaatccaaccaggcgtacaaca-3' |
| 377-379 | a100g_a101c_a103g_a104c_c106g_  5'-cgtaaagagtatgtgttgtacgcctggttgcagctgctctcacaacatccaaaataaactcatcttc-3'  5'-gaagatgagtttattttggatgttgtgagagcagctgcaaccaggcgtacaacacatactctttacg-3' |
| 380-382 | a109g_a112g_g113c_c115g_g116c_  5'-ggatatttcatcgtaaagagtatgtgttgtagccgcggctggattttttctcacaacatccaaaataaac-3'  5'-gtttattttggatgttgtgagaaaaaatccagccgcggctacaacacatactctttacgatgaaatatcc-3’ |
| 383-385 | a118g_a121g_c124g_a125c_  5'-taatgggatatttcatcgtaaagagtagctgctgcacgcctggttggattttttctcacaac-3'  5'-gttgtgagaaaaaatccaaccaggcgtgcagcagctactctttacgatgaaatatcccatta-3' |
| 386-388 | a127g_c130g_t131c_t133g_a134c_  5'-gttaggcacataatgggatatttcatcggcagcagcatgtgttgtacgcctggttggattttttctc-3'  5'-gagaaaaaatccaaccaggcgtacaacacatgctgctgccgatgaaatatcccattatgtgcctaac-3' |
| 389-391 | \| a137c_a140c_a142g_t143c_  5'-ggcgtacaacacatactctttacgctgcagcatcccattatgtgcctaaccacac-3'  5'-gtgtggttaggcacataatgggatgctgcagcgtaaagagtatgtgttgtacgcc-3' \| \| --- \| |
| 392-394 | t145g_c148g_a149c_t151g_a152c_  5'-cccgtgtggttaggcacagcagcggctatttcatcgtaaagagtatgtgttgtacgcctgg-3'  5'-ccaggcgtacaacacatactctttacgatgaaatagccgctgctgtgcctaaccacacggg-3' |
| 395-397 | t155c_c157g_a160g_a161c_  5'-taatagaattacccgtgtgggcagccgcataatgggatatttcatcgtaaagagtatgtg-3'  5'-cacatactctttacgatgaaatatcccattatgcggctgcccacacgggtaattctatta-3' |
| 398-400 | c163g_a164c_a166g_g170c_  5'-ctaaatcggtgcctaatagaattagccgcggcgttaggcacataatgggatatttc-3'  5'-gaaatatcccattatgtgcctaacgccgcggctaattctattaggcaccgatttag-3' |
| 401-403 | a172g_a173c_t175g_a178g_t179c_  5'-aaagatagactctaaatcggtgcctagcagcagcacccgtgtggttaggcacataatggg-3'  5'-cccattatgtgcctaaccacacgggtgctgctgctaggcaccgatttagagtctatcttt-3' |
| 404-406 | a181g_g182c_c184g_a185c_c187g_g188c_  5'-tctagtcttttggaaagatagactctaaatgcggccgcaatagaattacccgtgtggttaggcacata-3'  5'-tatgtgcctaaccacacgggtaattctattgcggccgcatttagagtctatctttccaaaagactaga-3' |
| 407-409 | t190g_t191c_a193g_g194c_t197c_  5'-acgtactctagtcttttggaaagataggctgcagctcggtgcctaatagaattacccgtgtg-3'  5'-cacacgggtaattctattaggcaccgagctgcagcctatctttccaaaagactagagtacgt-3' |

**Supplementary Table 4. 50:50 Primers for SHY->AAA in the endogenous *RAP1***

| 50 forward primer | GTGAGAAAAAATCCAACCAGGCGTACAACACATACTCTTTACGATGAAATAGCCGCTGCTGTGCCTAACCACACGGGTAATTCTATTAGGCACCGATTTAGAGTCTATCTGCTGGCTTAACTATGCGGCATCAGA |
| --- | --- |
| 50 reverse primer | AGATAGACTCTAAATCGGTGCCTAATAGAATTACCCGTGTGGTTAGGCACGTGCGGTATTTCACACCGCAGGG |
| SHY check F 21mer | TTACGATGAAATAGCCGCTGC |
| WT check F 21mer | TTACGATGAAATATCCCATTA |
| *RAP1* internal R | CGGTCCCCTACGGCTTTGGGT |

**Supplementary Table 5. Primers used for RT-qPCR**

| *ENO2* F | ACAACGTCATTGCTGCTGCT |
| --- | --- |
| *ENO2* R | TCAGCGGTTTGAATGTTTGG |
| *RPS5* F | TTGACTGACCAAAACCCAATCCA |
| *RPS5* R | CACCGACTCTGGTGGTGTCTTCT |
| *SPC42* F | AAGAGCTGCAAAGCATGATGGAC |
| *SPC42* R | GACTGGATTGGGAAGAATGACGA |

For a full list of RT-qPCR and ChIP-qPCR primers, see Platt et al., 2013.

**Supplementary Table 6. Oligonucleotides used for EMSA**

| TeloA-F | GCCGCACACCCACACACCAGTG |
| --- | --- |
| TeloA-R | CACTGGTGTGTGGGTGTGCGGC |
| *TEF2*-F | TGTTGCACCCACACATTTA |
| *TEF2*-R | TAAATGTGTGGGTGCAACA |
| *GAC1*-F | AATAATACATCACATTAACTGTCTATAAGAGGCTGGTAC |
| *GAC1*-R | GTACCAGCCTCTTATAGACAGTTAATGTGATGTATTATT |
